# Meaning in life in the Federal Republic of Germany: results of a representative survey with the Schedule for Meaning in Life Evaluation (SMiLE)

**DOI:** 10.1186/1477-7525-5-59

**Published:** 2007-11-22

**Authors:** Martin J Fegg, Mechtild Kramer, Claudia Bausewein, Gian D Borasio

**Affiliations:** 1 Interdisciplinary Center for Palliative Medicine, Ludwig-Maximilians-University, Marchioninistrasse 15, 81371 Munich, Germany

## Abstract

**Background:**

The construct "meaning-in-life" (MiL) has recently raised the interest of clinicians working in psycho-oncology and end-of-life care and has become a topic of scientific investigation. Difficulties regarding the measurement of MiL are related to the various theoretical and conceptual approaches and its inter-individual variability. Therefore the "Schedule for Meaning in Life Evaluation" (SMiLE), an individualized instrument for the assessment of MiL, was developed. The aim of this study was to evaluate MiL in a representative sample of the German population.

**Methods:**

In the SMiLE, the respondents first indicate a minimum of three and maximum of seven areas which provide meaning to their life before rating their current level of importance and satisfaction of each area. Indices of total weighting (IoW, range 20–100), total satisfaction (IoS, range 0–100), and total weighted satisfaction (IoWS, range 0–100) are calculated.

**Results:**

In July 2005, 1,004 Germans were randomly selected and interviewed (inclusion rate, 85.3%). 3,521 areas of MiL were listed and assigned to 13 a-posteriori categories. The mean IoS was 81.9 ± 15.1, the mean IoW was 84.6 ± 11.9, and the mean IoWS was 82.9 ± 14.8. In youth (16–19 y/o), "friends" were most important for MiL, in young adulthood (20–29 y/o) "partnership", in middle adulthood (30–39 y/o) "work", during retirement (60–69 y/o) "health" and "altruism", and in advanced age (70 y/o and more) "spirituality/religion" and "nature experience/animals".

**Conclusion:**

This study is a first nationwide survey on individual MiL in a randomly selected, representative sample. The MiL areas of the age stages seem to correspond with Erikson's stages of psychosocial development.

## Background

The concept of "meaning-in-life" (MiL) has recently stimulated the interest of clinicians and researchers working in psycho-oncology and end-of-life care. Moadel et al. [1] surveyed cancer patients and assessed their most important needs: 40% of the patients indicated a need for help in discovering meaning in their life. Meier et al. [2] found that 47% of the physicians who had granted at least one request for assisted suicide cited the patients' "loss of meaning in their lives" as a reason for the request. Meaning-Centered Group Psychotherapy was developed to help patients with advanced cancer to sustain or enhance a sense of meaning, peace and purpose in their lives [3].

The Austrian psychiatrist Victor Frankl [4] who had a personal history as a survivor of the Nazi concentration camps, developed the so-called logotherapy. He defined "meaning" as the manifestation of values, which are based on (i) creativity (e.g. work, deeds, dedication to causes), (ii) experience (e.g. art, nature, humor, love, relationships, roles), and (iii) attitude (one's attitude toward suffering and existential problems).

The different questionnaires developed so far to assess MiL [5-22] measure the intensity, but tend to neglect the content of the reported meanings, which vary from person to person and from situation to situation [23,24]. Since measurement of MiL based on standardized models and pre-selected domains may not provide a fully adequate representation of this highly individual construct, the "Schedule for Meaning in Life Evaluation" (SMiLE), based on a methodology utilized in quality of life (QoL) research, has been developed [25].

In QoL assessment, researchers faced similar problems, i.e. how to measure a highly individual concept, which is difficult to operationalize from a methodological point of view [24]. O'Boyle et al. therefore developed the "Schedule for the Evaluation of Individual Quality of Life – Direct Weighting" (SEIQoL-DW, [26,27]). In the SEIQoL-DW, the respondent indicates domains of individual QoL and rates their relative importance and satisfaction with each domain. The SMiLE was developed analogously to the SEIQoL methodology with the aim to provide an individualized assessment of MiL [25].

### Objectives

The objective of this study was to evaluate individual MiL in a representative sample of the German population to gather data for future comparisons with cancer and palliative care patients. More specifically, the study aimed (i) to evaluate and categorize individually important MiL areas, and (ii) to examine differences between sociodemographic parameters and MiL.

## Methods

### Study design

The design of the study was cross-sectional. In July 2005, a representative nationwide German sample was interviewed with assistance of Forsa, a German Social Research Institute. The survey consisted of computer assisted telephone interviews. All telephone numbers, comprising published and unpublished numbers, were randomly selected. To obtain a random sample, the member of the household who most recently had birthday was asked to participate. Appointments were made if the target person was not available or requested later completion. All 50 interviewers were well-experienced in telephone interviews and received a written, standardized protocol of the SMiLE method. All German speaking individuals, aged 16 years and older, living in private households equipped with a telephone, were eligible for the study.

Sociodemographic data consisted of age, gender, marital status, education, employment, household net income, residence, and federal state. The federal states were classified according to ACNielsen [28]: 1) Hamburg, Bremen, Schleswig-Holstein, Lower Saxony; 2) North Rhine-Westphalia; 3a) Hesse, Rhineland-Palatinate, Saarland; 3b) Baden-Wuerttemberg; 4) Bavaria; 5) Berlin; 6) Mecklenburg-Western Pommerania, Brandenburg, Saxony-Anhalt; and 7) Thuringia, Saxony.

### The Schedule for Meaning in Life Evaluation (SMiLE)

The SMiLE is an individualized measure of MiL which was developed in accordance to the recommendations of the Scientific Advisory Committee of the Medical Outcomes Trust [29].

#### Step 1 (area listing)

The respondents indicate a minimum of three and maximum of seven areas (n = number of areas) which provide meaning to their life in their current situation.

#### Step 2 (weighting)

The importance of each area (w_1_...w_n_; 3 ≤ n ≤ 7) is rated with a five-point adjectival scale, ranging from 1 "somewhat important" to 5 "extremely important".

#### Step 3 (level of satisfaction)

The respondents rate their current level of satisfaction with each area (s_1_...s_n_; with 3 ≤ n ≤ 7) on a seven-point Likert scale, ranging from -3 "very unsatisfied" to +3 "very satisfied".

The **Index of Weighting (IoW) **indicates the mean weighting of the MiL areas (range, 20–100, with higher scores reflecting higher weights). Since the scale starts with "somewhat important", the floor is set to 20 instead of 0.

IoW=20∘wgesn;Wges=∑i=1nwi.
 MathType@MTEF@5@5@+=feaafiart1ev1aaatCvAUfKttLearuWrP9MDH5MBPbIqV92AaeXatLxBI9gBaebbnrfifHhDYfgasaacPC6xNi=xI8qiVKYPFjYdHaVhbbf9v8qqaqFr0xc9vqFj0dXdbba91qpepeI8k8fiI+fsY=rqGqVepae9pg0db9vqaiVgFr0xfr=xfr=xc9adbaqaaeGacaGaaiaabeqaaeqabiWaaaGcbaGaemysaKKaem4Ba8Maem4vaCLaeyypa0JaeGOmaiJaeGimaaJaeSigI8wcfa4aaSaaaeaacqWG3bWDdaWgaaqaaiabdEgaNjabdwgaLjabdohaZbqabaaabaGaemOBa4gaaOGaei4oaSJaee4vaC1aaSbaaSqaaiabbEgaNjabbwgaLjabbohaZbqabaGccqGH9aqpdaaeWbqaaiabdEha3naaBaaaleaacqWGPbqAaeqaaaqaaiabdMgaPjabg2da9iabigdaXaqaaiabd6gaUbqdcqGHris5aOGaeiOla4caaa@4E13@

The **Index of Satisfaction (IoS) **indicates the mean satisfaction or dissatisfaction with the individual MiL areas (range, 0–100, with higher scores reflecting higher satisfaction). To obtain a clear index varying from 0 to 100, the satisfaction ratings s_i _are recalculated (s'_i_). "Very unsatisfied" (s_i _= -3) is set to s'_i _= 0 and "very satisfied" (s_i _= +3) is set to s'_i _= 100 with the levels of 16.7, 33.3, 50, 66.7, and 83.3 in between.

IoS=∑i=1ns'in.
 MathType@MTEF@5@5@+=feaafiart1ev1aaatCvAUfKttLearuWrP9MDH5MBPbIqV92AaeXatLxBI9gBaebbnrfifHhDYfgasaacPC6xNi=xI8qiVKYPFjYdHaVhbbf9v8qqaqFr0xc9vqFj0dXdbba91qpepeI8k8fiI+fsY=rqGqVepae9pg0db9vqaiVgFr0xfr=xfr=xc9adbaqaaeGacaGaaiaabeqaaeqabiWaaaGcbaGaemysaKKaem4Ba8Maem4uamLaeyypa0tcfa4aaSaaaeaadaaeWbqaaiabdohaZjabcEcaNmaaBaaabaGaemyAaKgabeaaaeaacqWGPbqAcqGH9aqpcqaIXaqmaeaacqWGUbGBaiabggHiLdaabaGaemOBa4gaaiabc6caUaaa@3E60@

In the **total SMiLE index (Index of Weighted Satisfaction, IoWS)**, the ratings for importance and satisfaction are combined (range, 0–100, with higher scores reflecting higher MiL).

IoWS=∑i=1n(wiwges∘s'i).
 MathType@MTEF@5@5@+=feaafiart1ev1aaatCvAUfKttLearuWrP9MDH5MBPbIqV92AaeXatLxBI9gBaebbnrfifHhDYfgasaacPC6xNi=xI8qiVKYPFjYdHaVhbbf9v8qqaqFr0xc9vqFj0dXdbba91qpepeI8k8fiI+fsY=rqGqVepae9pg0db9vqaiVgFr0xfr=xfr=xc9adbaqaaeGacaGaaiaabeqaaeqabiWaaaGcbaGaemysaKKaem4Ba8Maem4vaCLaem4uamLaeyypa0ZaaabCaeaadaqadaqaaKqbaoaalaaabaGaem4DaC3aaSbaaeaacqWGPbqAaeqaaaqaaiabdEha3naaBaaabaGaem4zaCMaemyzauMaem4CamhabeaaaaGccqWIyiYBcqWGZbWCcqGGNaWjdaWgaaWcbaGaemyAaKgabeaaaOGaayjkaiaawMcaaaWcbaGaemyAaKMaeyypa0JaeGymaedabaGaemOBa4ganiabggHiLdGccqGGUaGlaaa@49DA@

Before completion of the SMiLE, participants are asked to rate their overall MiL satisfaction on a seven-point Likert scale, ranging from -3 "very unsatisfied" to +3 "very satisfied" (MiL_NRS).

The **psychometric properties of the SMiLE **were evaluated in a study [25] with 599 students of the Ludwig-Maximilians-University, Munich, and the Royal College of Surgeons, Dublin (response rate, 95.4%). Mean IoW was 85.7 ± 9.4, mean IoS was 76.7 ± 14.3, and mean IoWS was 77.7 ± 14.2. Test-retest reliability of the IoWS was r = 0.72 (p < .001), with 85.6% of all areas listed again after a test-retest period of seven days. Convergent validity was evaluated with the Purpose in Life test [5] (r = 0.48, p < .001), the Self-Transcendence Scale [30] (r = 0.34, p < 001), and a general NRS on MiL (r = 0.53, p < .001). The psychometrics of the SMiLE were reported according to the recommendations of the Scientific Advisory Committee of the Medical Outcome Trust [29].

### Statistical Analysis

Multifactorial analyses of variance (F-test) were performed for the outcome scores (IoS, IoW, IoWS, MiL_NRS) to control for potential confounders. Independent variables included age, gender, marital status, education, employment, household net income, residence, and federal states. To identify differences in the likelihood of listing a specific MiL area, parameter estimators (B) of the multifactorial analyses of variance and Chi-Square tests were used. Odds ratios (ORs) with 95% confidence intervals (CIs) were calculated to describe the relation between respondents with the most and least likelihood of listing an area.

A posteriori binary cluster analyses (linkage between groups, phi-4-point correlation) were calculated to facilitate the categorization of these MiL areas.

All p-values are Bonferroni corrected. Differences were considered to be statistically significant at p = .05. Statistical tests were performed with the Statistical Package for Social Sciences (SPSS), version 13.0.

## Results

### Participation in the study

The mean response rate in telephone surveys in Germany is around 75% (Forsa, personal communication, March 27, 2006). 1,004 individuals were interviewed, 148 participants were excluded because they did not list the required number of at least three areas of MiL. In total, 856 individuals completed the interview (inclusion rate, 85.3%).

### Respondent's characteristics

Table 1 provides an overview of the respondents' characteristics.

**Table 1:** Respondents' characteristics (n = 856).

		n	%
Age	16–19 years	51	5.9
	20–29 years	124	14.5
	30–39 years	164	19.1
	40–49 years	161	18.8
	50–59 years	119	14.0
	60–69 years	127	14.8
	70 years and above	110	12.9

Gender	Male	423	49.4
	Female	433	50.6

Marital status	Single	269	31.6
	Married	428	50.1
	Divorced/Separated	90	10.5
	Widowed	67	7.8

Education	Elementary school	205	25.4
	Secondary school	286	35.4
	High school	317	39.2

Occupational status	Employed	436	51.0
	Unemployed	420	49.0

Household net income (per month)	999 € or less	82	12.2
	1.000 – 1.999 €	231	34.4
	2.000 – 2.999 €	159	23.7
	3.000 € and more	200	29.8

Residence	Less than 5.000 inhabitants	148	17.3
	5.000 – 9.999	149	17.4
	10.000 – 49.999	241	28.1
	50.000 – 99.999	69	8.1
	100.000 and more	249	29.1

### Item characteristics

In total, 3,521 areas of MiL were listed by the respondents. All open answers were transcribed and assigned to 39 a-posteriori MiL categories by two independent raters (MJF, MK). Afterwards, binary cluster analyses were calculated to include areas with frequencies ≥3%. The results of the cluster analyses led to the following thirteen categories that represent the different MiL areas:

1. Altruism (altruism, helping others, readiness to help, volunteer work).

2. Animals/Nature (animals, fond of animals, nature, nature-love, pets).

3. Family (children, family, grandchildren, parents, relatives, siblings).

4. Financial Security (finances, income, money, property, prosperity).

5. Friends/Acquaintances (acquaintances, friends, neighbors, human/social relations).

6. Health (health, physical well-being).

7. Hedonism (consumption, having fun, pleasure).

8. Home/Garden (apartment, gardening, home, housing).

9. Leisure Time (cinema, culture, drama, hobbies, holiday, music, sport).

10. Occupation/Work (job, occupation, professional success, work, working place).

11. Partnership (love, marriage, partner, partnership).

12. Psychological Well-Being (harmony, luck, mental satisfaction/well-being).

13. Spirituality/Religion (church, faith, God, Jesus, religion, spirituality).

Table 2 shows weight and satisfaction of the listed MiL areas. In median, 4 areas of MiL were listed by the respondents (3 areas, 43.3%; 4 areas, 27.7%; 5 areas, 14.3%; 6 areas, 6.9%; 7 areas, 7.8%).

**Table 2:** Areas of MiL listed by the respondents (n = 856). Included are number and percentage of the listings as well as mean and standard deviation (SD) of the importance and satisfaction ratings.

			w_i_	s_i_
	n	%	Mean ± SD	Mean ± SD

Family	708	82.7	4.7 ± 0.6	2.3 ± 0.9
Work	463	54.1	3.9 ± 0.9	1.4 ± 1.6
Leisure time	350	40.9	3.5 ± 1.0	1.6 ± 1.4
Friends	340	39.7	4.3 ± 0.8	2.2 ± 1.0
Health	276	32.2	4.8 ± 0.4	1.8 ± 1.5
Partnership	233	27.2	4.7 ± 0.6	2.4 ± 1.1
Finances	124	14.5	3.6 ± 1.1	1.0 ± 1.8
Home/Garden	81	9.5	3.5 ± 1.1	2.0 ± 1.1
Spirituality	80	9.4	4.4 ± 0.9	2.4 ± 0.9
Animals/Nature	79	9.2	4.1 ± 0.9	2.3 ± 1.0
Hedonism	41	4.7	4.2 ± 0.9	1.9 ± 1.3
Altruism	39	4.6	3.8 ± 0.8	2.1 ± 0.9
Well-Being	37	4.3	4.4 ± 0.8	1.8 ± 1.3

The study subjects were most satisfied with partnership and spirituality and least satisfied with work and finances. Health, partnership, and family were rated as most important for MiL, home/garden and leisure time were least important.

In multifactorial analyses of variance, significant influences were found for age, gender, education, household net income, residence, and federal states. No significant influences were found for marital status and employment.

### Age

**Figure 1:**
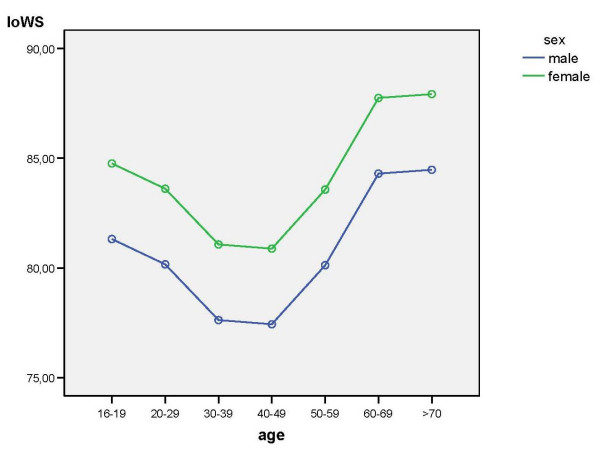
Results of the multifactorial analysis with the effects of age and gender on IoWS.

**Figure 2:**
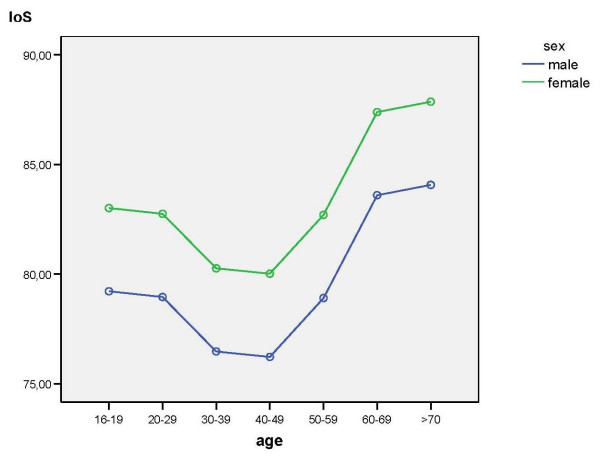
Results of the multifactorial analysis with the effects of age and gender on IoS.

The IoWS, IoS, and MiL_NRS were influenced by age (df = 6, p_IoWS _= .01, p_IoS _= .006, p_MiL_NRS _= .001). Figure 1, 2, 3 show the significant effects of age and gender on these outcome scores.

**Figure 3:**
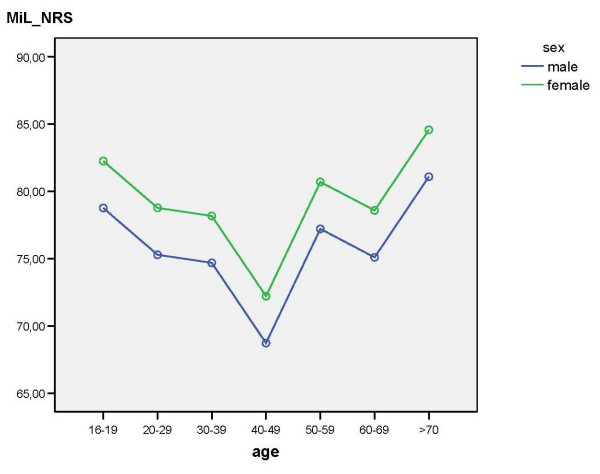
Results of the multifactorial analysis with the effects of age and gender on MiL_NRS.

In the listed areas, differences were found for altruism (p = .002), animals/nature (p = .03), friends (p < .001), health (p < .001), partnership (p < .001), spirituality (p < .001), and work (p < .001). Post-hoc tests showed that individuals aged 16–19 years were most likely to list friends (compared to ≥70, OR 11.6, CI 5.2–25.6), 20–39 y/o individuals listed partner (compared to ≥70, OR 4.8, CI 2.5–9.2), 30–39 y/o work (compared to ≥70, OR 24.4, CI 11.9–52.6), 60–69 y/o health (compared to 16–19, OR 38.2, CI 5.1–285.2) and altruism (compared to 20–29, OR 16.5, CI 2.1–126.7), and individuals aged 70 years and above were most likely to list animals/nature (compared to 16–19, OR 5.4, CI 1.2–24.0) and spirituality (compared to 30–39, OR 7.7, CI 3.0–19.4).

### Gender

The IoWS, IoS, IoW, and MiL_NRS are influenced by gender (df = 1, p_IoWS _= .003, p_IoS _= .001, p_IoW _< .001, p_MiL_NRS _= .02). Males scored lower on all outcome scores (B_IoWS _= -3.4, B_IoS _= -3.8, B_IoW _= -3.9, B_MiL_NRS _= -3.5).

Women were more likely to list animals/nature (OR 2.9, CI 1.8–4.9; p < .001), family (OR 3.0, CI 2.0–4.4; p < .001), and health (OR 2.3, CI 1.7–3.1; p < .001).

### Education

MiL_NRS was influenced by education (df = 2, p = .002). Individuals with high school degree were more satisfied than individuals with an elementary school degree (B = -2.3) or a second school degree (B = -5.8).

In the listed areas, differences were found for finances (p = .03), health (p < .001), leisure time (p = .004), spirituality (p = .02), and work (p < .001). Post-hoc tests showed that individuals with an elementary school degree were most likely to list finances (compared to high school, OR 2.6, CI 1.5–4.3, no influence of net income) and health (compared to high school, OR 2.6, CI 1.8–3.8). Individuals with high school degree were most likely to list leisure time (compared to elementary school, OR 2.3, CI 1.6–3.3), spirituality (compared to elementary school, OR 1.8, CI 1.0–3.3), and work (compared to elementary school, OR 2.0, CI 1.4–2.9).

### Household net income

MiL_NRS was influenced by household net income (df = 3, p = .004). Subjects with the highest income (>3,000€) were most satisfied with their MiL compared to respondents with lower income (2,000–3,000€: B = -1.9; 1,000–2,000€: B = -3.1; <1,000€: B = -9.9).

In the listed areas, a difference was found for work (p = .04). A post-hoc test showed that individuals with the highest net income (>3,000€) were most likely to list work (compared to < 1,000€, OR 1.8, CI 1.0–3.0).

### Residence

The IoWS and IoS were influenced by residence (df = 4, p_IoWS _= .03, p_IoS _= .02). Subjects living in rural areas (<5,000 inhabitants) were most satisfied (B_IoWS _= 5.0, B_IoS _= 5.3), subjects from big cities were least satisfied (>100,000: B = 0; 5–10,000: B_IoWS _= 4.1, B_IoS _= 4.1; 10–50,000: B_IoWS _= 2.8, B_IoS _= 2.9; 50–100,000: B_IoWS _= 3.4, B_IoS _= 3.2).

In the listed areas, no significant differences were found.

### Federal states

MiL_NRS was influenced by federal states (df = 2, p < .001): Inhabitants of the German South-West (Nielsen 2, 3a, 3b, 4; B = 7.3) were most satisfied, followed by the German North (Nielsen 1; B = 4.8). The German East (Nielsen 5, 6, 7; B = 0) was least satisfied.

In the listed areas, a difference was found for home/garden (p = .004). Post-hoc tests showed that individuals living in the German East were more likely to list home/garden (compared to South-West, OR 3.0, CI 1.8–5.0).

## Discussion

This study is a first nationwide survey on MiL in a randomly selected, representative general population with a respondent-generated MiL instrument.

The data presented here may be a useful basis for comparisons in future studies with physically or mentally ill patients, and also for the evaluation of meaning-based interventions recently developed in end-of-life care [3]. Compared to the SEIQoL-DW (measuring QoL), the SMiLE (measuring MiL) has a simpler weighting procedure (adjectival scale vs. Pie-Chart-Technique). In a previous study [25], university students were asked how they differentiate between QoL and MiL: they stated that MiL was related for them to spirituality and self-transcendence, whereas QoL reflected their current status of subjective well-being. The "idiographic" approach in both instruments (SEIQoL-DW, SMiLE) responds to general and philosophical arguments against standardized ("nomothetic") QoL and MiL measurement: these are highly individual constructs which need a subjective and individualized approach [24]. However, statistical comparisons are more difficult with idiographic measures.

Thirteen categories were found to represent 2,851 of 3,521 areas of MiL (81.0%) listed by the respondents. Health, partnership, and family were rated as most important, home/garden and leisure time were least important. Subjects were most satisfied with partnership and spirituality, and least satisfied with work and finances.

The categories are similar to findings of earlier studies. The areas of Ebersole [31] consist of activities, beliefs, growths, healths, life work, obtainings, pleasures, relationships, and services. Reker & Wong [32] found altruism, meeting basic needs (e.g. food, shelter, safety), creative work, enduring values/ideals, legacy, leisure activities/hobbies, personal achievement, personal growth, personal relationships, religion, social/political activism, and traditions/culture.

The well-known "midlife crisis" is reflected by the finding that individuals aged 40–49 years were least satisfied with their MiL. The different MiL areas in the age stages seem to correspond with Erikson's last four stages in psychosocial development [33]. In youth (16–19 years, psychosocial stage – "Identity vs. Role Confusion"), friends are most important. In young adulthood (20–29 years, psychosocial stage – "Intimacy and Solidarity vs. Isolation"), partnership is getting more and more important. In middle adulthood (30–39 years, psychosocial stage – "Generativity vs. Self-Absorption and Stagnation"), work is more likely to be listed and the overall MiL is decreasing. After success in procreation and attainment of solid job positions, health and altruism are becoming important during retirement (60–69 years, psychosocial stage – "Ego Integrity vs. Despair"). In advanced age (70 years and above, psychosocial stage – "Ego Integrity vs. Despair"), spirituality/religion and experience of nature/animals are getting more and more important and support overall MiL satisfaction.

The Eriksonian approach is life-span oriented: all stages are marked by a specific conflict. The individual has to learn to hold both extremes of the life-stage challenges in tension with one another [33]. Future studies are necessary to enhance empirical evidence of this model and to improve the integration into life-span oriented psychological interventions.

In general, women were more satisfied with their MiL and listed more important areas. Furthermore, they focused on animals/nature, family, and health. Value researchers found that women emphasize expressive-communal values (e.g. creativity, nature experience), while men emphasize instrumental values (e.g. job, achievement, power) [34].

Subjects in rural areas and urban agglomerations were more satisfied in MiL compared to subjects living in urbanized areas or cities. This is supported by the General Social Survey (GSS) which found that rural residents had significantly higher levels of family life satisfaction and community satisfaction [35].

Inhabitants of the affluent German South-West (Baden-Wuerttemberg, Bavaria, Hesse/Rhineland-Palatinate/Saarland, and North Rhine-Westphalia) were most satisfied with their overall MiL. Other surveys have also found that residents of West Germany were more satisfied in almost all life domains with the differences to East Germany becoming smaller [36]. The "Perspektive Deutschland" [37], an online survey of public opinion with more than 510,000 participants, found that Bavaria and Baden-Wuerttemberg had the highest satisfaction scores in Germany but East Germany's satisfaction is rising.

### Limitations

The advantage of surveys using telephone interviews is the cost-effectiveness and high response rate but the precision depends on the training of the interviewers. The research institute was well-experienced and all interviewers received a written, standardized protocol of the SMiLE method. Nevertheless, face-to-face interviews would have increased the survey's validity.

The respondent generated listings were assigned to a-posteriori categories. It is possible that not all listings were identified correctly. Sometimes it was difficult to differentiate between nature vs. garden and spirituality vs. psychological well-being. Additionally, assessment of individual meanings of the listed areas is limited in telephone interviews. For example, many respondents list "family" as a cue label, but it can have various meanings for the individual: feeling secure, taking care of the children, loving and being loved, or pleasure in social activities of the family. For further understanding, it will be necessary to obtain in-depth descriptions of what is meant by the cue labels, e.g. using qualitative research designs [23].

## Conclusion

This study investigated MiL in a representative survey of the German population with an individualized assessment tool, the Schedule for Meaning in Life Evaluation (SMiLE). In the open answers, 13 MiL categories were found. Multifactorial analyses of variance showed significant influences of sociodemographic parameters on the listed areas and the outcome scores of the SMiLE. The likelihood of MiL areas listed during the age stages of this survey seem to correspond with Erikson's phases of the psychosocial development.

Many existing MiL questionnaires are based on the theoretical background of the researchers [38]. An advantage of the SMiLE is to be a non-theoretically driven assessment tool. The subjects themselves nominate areas which are important to their individual MiL. Since a consensus in the definition of MiL is still missing [39], an attempt to define MiL for the individualized approach may read as follows (paraphrasing O'Boyle [24]): "Meaning in life is what the individual says it is".

## Abbreviations

IoW Index of Weighting

IoS Index of Satisfaction

IoWS Index of Weighted Satisfaction

MiL Meaning in Life

MiL_NRS Numeric Rating Scale on MiL satisfaction

n numbers of MiL areas listed

QoL Quality of Life

s_1_...s_n _satisfaction with each MiL area

SEIQoL Schedule for the Evaluation of Individual Quality of Life

SMiLE Schedule for Meaning in Life Evaluation

w_1_...w_n _weighting/importance of each MiL are

## Competing interests

The author(s) declare that they have no competing interests.

## Authors' contributions

MF designed the study, analyzed the data, interpreted the results and wrote the manuscript. MK, CB and GDB were involved in the planning of the design, the interpretation of the results and the writing of the manuscript. All authors read and approved the final manuscript.
